# A comparative study of drift diffusion and linear ballistic accumulator models in a reward maximization perceptual choice task

**DOI:** 10.3389/fnins.2014.00148

**Published:** 2014-08-05

**Authors:** Stephanie Goldfarb, Naomi E. Leonard, Patrick Simen, Carlos H. Caicedo-Núñez, Philip Holmes

**Affiliations:** ^1^HRL Laboratories, Center for Neural and Emergent SystemsMalibu, CA, USA; ^2^Mechanical and Aerospace Engineering, Princeton UniversityPrinceton, NJ, USA; ^3^Program in Applied and Computational Mathematics, Princeton UniversityPrinceton, NJ, USA; ^4^Princeton Neuroscience Institute, Princeton UniversityPrinceton, NJ, USA; ^5^Neuroscience Department, Oberlin CollegeOberlin, OH, USA

**Keywords:** drift diffusion model, linear ballistic accumulator model, reward maximization, optimal performance theory

## Abstract

We present new findings that distinguish drift diffusion models (DDMs) from the linear ballistic accumulator (LBA) model as descriptions of human behavior in a two-alternative forced-choice reward maximization (Rmax) task. Previous comparisons have not considered Rmax tasks, and differences identified between the models' predictions have centered on practice effects. Unlike the parameter-free optimal performance curves of the pure DDM, the extended DDM and LBA predict families of curves depending on their additional parameters, and those of the LBA show significant differences from the DDMs, especially for poorly discriminable stimuli that incur high error rates. Moreover, fits to behavior reveal that the LBA and DDM provide different interpretations of behavior as stimulus discriminability increases. Trends for threshold setting (caution) in the DDMs are consistent between fits, while in the corresponding LBA fits, thresholds interact with distributions of starting points in a complex manner that depends upon parameter constraints. Our results suggest that reinterpretation of LBA parameters may be necessary in modeling the Rmax paradigm.

## 1. Introduction

Among the many models proposed to describe decision tasks, leaky competing accumulators (LCAs) (Usher and McClelland, 2001) and drift diffusion models (DDMs) e.g., Ratcliff and Rouder (1998) have been especially prominent. More recently the linear ballistic accumulator (LBA) (Brown and Heathcote, 2008) was introduced as a conceptually simpler alternative to DDMs. All these models employ drift terms that describe mean rates of evidence accumulation, thresholds that signal decision times when crossed, and sources of variability, either within or across trials. All have been validated against particular behavioral data, but since they differ in structure, number of parameters, and the manner in which variability enters, they may suggest different processing mechanisms [although the DDM can be derived from the LCA under certain conditions (Bogacz et al., 2006)]. It is therefore of interest to compare their accounts of given data sets.

The comparative study of Donkin et al. (2011) revealed few differences between the abilities of the LBA and DDM to fit and predict behavioral data. However, an earlier comparison of DDM fits to simulated data from LBA, DDM, and LCA found that DDM and LCA parameters correlated in a one-to-one manner, but those of LBA and DDM did not (van Ravenzwaaij and Oberauer, 2009). Subsequently, differences in drift rates, non-decision times and caution parameters were found in many-parameter fits of practice effects (Heathcote and Hayes, 2012), but these differences were not connected to optimal theories of performance in perceptual choice tasks. LBA fits were not included in a substantial recent paper (Teodorescu and Usher, 2013) that compared several race and LCA models. Nor have the LBA and DDMs been compared for reward maximization (Rmax) tasks in which participants have learned strategies and apply task-based knowledge to optimize performance.

In Rmax tasks participants are instructed to adopt a strategy that yields maximum rewards, and are given a fixed time interval to complete each block of trials, during which they may attempt the task as many times as they wish, as detailed in section 2.3. Task difficulty is held constant within a block but varied between blocks. In the two-alternative forced-choice (2AFC) task from which data is analyzed here, visual moving dots stimuli were used and task difficulty was adjusted via motion coherence (Balci et al., 2011). Depending on difficulty, a participant may attempt the task few times, slowly and cautiously, or she may work faster but more carelessly. The 2AFC Rmax task performance and model fits have been tested against the DDM, and DDM fits have been shown to describe a speed-accuracy tradeoff quite close to that of high performing participants (Bogacz et al., 2006; Simen et al., 2009; Bogacz et al., 2010; Balci et al., 2011). However, Rmax task performance and fits have not previously been compared across models.

Here we compare the LBA, which represents evidence in favor of two or more options, with the pure and extended (Ratcliff, e.g., Ratcliff and Rouder, 1998) DDMs, which assess differences of evidence between options. In the LBA two drift rates, believed to be correlated with neural activity (e.g., Gold and Shadlen, 2000, 2001; Gold et al., 2008), represent preferences for each of the two options; in the DDMs, a single drift rate represents the difference between these preferences.

For both the DDMs and the LBA models, thresholds, also called caution parameters (Donkin et al., 2011), set a level of accumulated activity at which a decision is made. Caution is key in setting the speed-accuracy tradeoff: high caution implies low speed and high accuracy and low caution implies high speed and low accuracy (Bogacz et al., 2006; Brown and Heathcote, 2008; Balci et al., 2011). For example, caution can explain the relatively slow response times of elderly individuals (Ratcliff et al., 2004). Caution can be experimentally manipulated by adjusting task difficulty from block to block, and optimal values of caution can be determined analytically for the pure DDM and numerically for the extended DDM and the LBA, as shown below in section 2.2.

An important difference between the DDMs and the LBA models is the treatment of variability. In the DDMs variability enters as additive Gaussian noise in the evidence accumulation dynamics during each individual trial. In the extended DDM, there are additional trial-to-trial variabilities in the starting point, in the drift rate of evidence accumulation, and in the non-decision time. In contrast, there is no additive noise in the LBA during individual trials, as implied by the adjectives “linear” and “ballistic.” Instead there is only trial-to-trial variability in the starting points and in the drift rates. Nonetheless, the LBA models can capture much of the same behavior as the extended DDM, and they do so with fewer parameters (Brown and Heathcote, 2008; Donkin et al., 2011).

Direct numerical comparisons of the role of caution in the models are straightforward. Parameters can be fit to participant behavior at each difficulty level for each model. For a given parameter set, changes in speed and accuracy of responses as caution is varied can be computed, and thus the value of the caution parameter that yields the highest reward rate can be determined for each difficulty level. An optimal speed-accuracy tradeoff for each model and participant can then be derived, assuming that caution is varied from one difficulty condition to the next.

The models can also be evaluated and compared by examining their predictions of optimal performance. For the pure DDM, a unique parameter-free Optimal Performance Curve (OPC) describes the relationship between error rate (ER) and a normalized decision time (DT), independent of model parameters (Bogacz et al., 2006). Parameterized families of OPCs may also be determined for the extended DDM, and as the values of the additional parameters (variances in drift rate, in starting point, and in non-decision time) become small, these curves approach that of the pure DDM (Bogacz et al., 2006). Like the extended DDM, the LBA does not predict unique OPCs, and our analysis of the LBA reveals a critical interaction between thresholds and variance in starting points.

While the DDMs and the LBA can reproduce key aspects of behaviors, the DDM fits suggest that participants are on average *least* cautious on the most difficult tasks, in which the optimal strategy is random guessing. In contrast, an LBA fit indicates that participants are on average *more* cautious on the most difficult tasks, and that they reduce variance in starting points as difficulty decreases. These differences between the DDM and the LBA predictions highlight the role of diffusive noise within trials in the DDMs, which is sacrificed for simplicity in the LBA model.

The paper is structured as follows. In section 2 we discuss our methods, describing the LBA model, the pure and extended DDMs, and parameter-fitting procedures. We review optimal performance theory for the pure and extended DDMs, develop analogous results for the LBA, propose an adapted LBA that decouples starting point from thresholds and compare model performances in the limit of large noise. Section 3 describes our results primarily in terms of parameter fits across subjects, and section 4 contains a discussion and directions for future work. Details of fits to individual participants are provided in the Supplementary Material.

## 2. Materials and methods

### 2.1. Drift diffusion and linear ballistic accumulation

**Figure 1:**
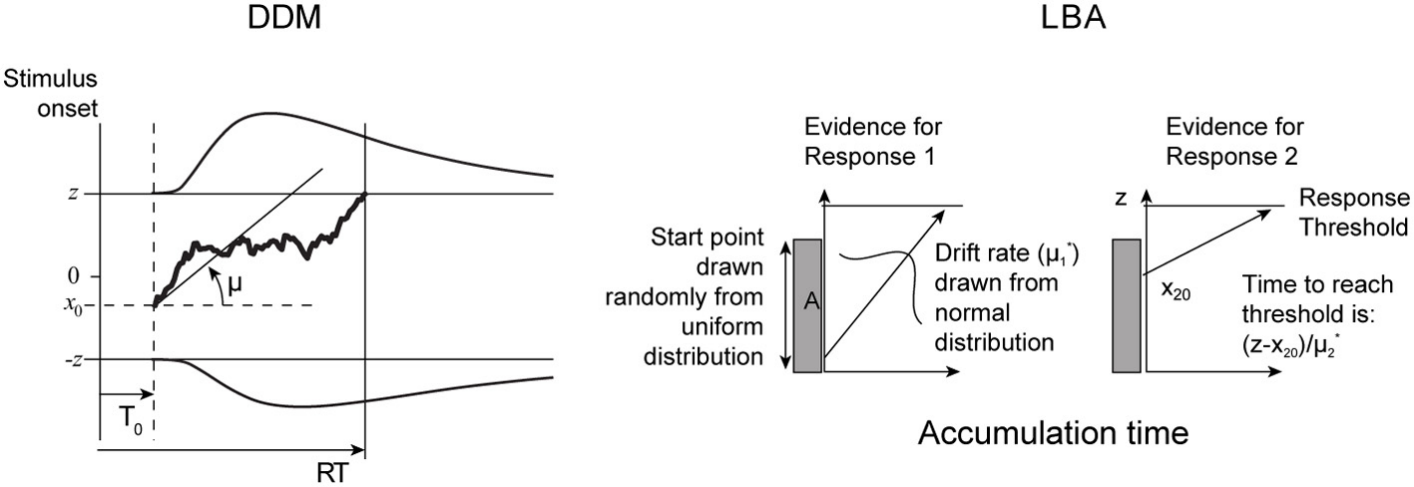
**Comparative illustration of pure DDM (left) and LBA (right) models**. See text for description of extended DDM and further details.

In this section we describe the pure and extended (Ratcliff) DDMs and the LBA models. The two models are illustrated in Figure 1.

#### 2.1.1. Pure drift diffusion model

In the pure DDM the difference in evidence for the two choices evolves according to the following scalar equation:

(1) $$dx=\mu dt+\sigma dW,x(0)=x_{0},$$

where *x*(*t*) is the aggregate evidence at time *t*, μ is the drift rate, σ is the diffusion rate, and *dt* and *dW* represent time and Wiener noise increments, respectively. Evidence accumulates noisily from the starting point *x*(0) = *x*_0_ at time *t* = 0 to the first time *t* = *T* at which *x*(*T*) = +*z* or −*z*. Without loss of generality, we assume that μ ≥ 0 (Bogacz et al., 2006). Thus, the two thresholds, +*z* and −*z*, respectively, correspond to selecting the correct and incorrect choices. We will refer to *z* interchangeably as the *threshold* or the *caution* (parameter); z can take any non-negative value.

Only four parameters are required to predict DT for Equation (1): μ, *x*_0_, σ, and *z*, and there are closed form analytical expressions for mean DT and ER (Bogacz et al., 2006):

(2) $$\text{DT}=\frac{z}{\mu }\tanh\text{​}\left(\frac{z\mu }{\sigma^{2}}\right)+\frac{2z}{\mu }\cdot \text{​}\left(\frac{1-\exp\text{​}\left(\frac{2x_{0}\mu }{\sigma^{2}}\right)}{\exp\text{​}\left(\frac{2z\mu }{\sigma^{2}}\right)-\exp\text{​}\left(-\frac{2z\mu }{\sigma^{2}}\right)}\right)-\frac{x_{0}}{\mu },$$

(3) $$\text{ER}=\frac{1}{1+\exp\text{​}\left(\frac{2z\mu }{\sigma^{2}}\right)}-\text{​}\left(\frac{1-\exp\text{​}\left(\frac{2x_{0}\mu }{\sigma^{2}}\right)}{\exp\text{​}\left(\frac{2z\mu }{\sigma^{2}}\right)-\exp\text{​}\left(-\frac{2z\mu }{\sigma^{2}}\right)}\right).$$

In addition, the pure DDM is augmented by a non-decision time parameter, *T*_0_, corresponding to non-decision processes. The estimated reaction time (RT) is the sum of the decision and non-decision times: RT = DT + *T*_0_.

Although our data were derived from unbiased stimuli, we allow non-zero starting points in order to make direct comparisons among all the models, since extended DDMs and LBAs use ranges of starting points.

#### 2.1.2. Extended drift diffusion model

In the extended (Ratcliff) DDM, evidence accumulation in each trial is governed by the same process as in the pure DDM, but with added variability in starting points, drift rate, and non-decision time:

(4) $$dx=\mu^{*}dt+\sigma dW,x(0)=x_{0}^{*},$$

where μ^*^, σ, *z*, x^*^_0_, and T^*^_0_, respectively represent the drift rate, diffusion rate, threshold, starting point, and non-decision time for a given trial. Evidence accumulation proceeds from the starting point *x*(0) = *x*_0_ at time *t* = 0 to the first time *t* = *T* at which *x*(*T*) = +*z* or −*z*. For each trial, μ^*^ is selected from 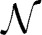 (μ, *s*^2^_μ_), x^*^_0_ is selected from 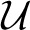 $\left(x_{0}-\frac{s_{x_{0}}}{2},x_{0}+\frac{s_{x_{0}}}{2}\right)$, and T^*^_0_ is selected from 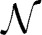 (*T*_0_, *s*^2^_*T*__0_), where 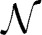 and 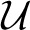 respectively denote Gaussian (normal) and uniform distributions, and μ, *s*_μ_, *s*_*x*_0__, *T*_0_, *s*_*T*_0__ are all non-negative constant scalars.

The additional variability in the model parameters from trial to trial augments the model's descriptive power. In particular, the extended DDM, unlike the pure DDM, can predict different RT distributions for correct and error trials, even with unbiased mean starting points. Prior work has suggested that the parameters new to the extended DDM sufficiently extend the descriptive capabilities of the DDM to merit the additional modeling cost (Ratcliff and Rouder, 1998; Ratcliff and Smith, 2004; Balci et al., 2011). However, analytical expressions for DT and ER analogous to Equations (2, 3) do not exist for the extended DDM. The extended DDM is frequently called the Ratcliff DDM due to a large body of work by Ratcliff to characterize it. Here the threshold *z* can assume any non-negative value outside the range of starting points (Tuerlinckx, 2004).

#### 2.1.3. Linear ballistic accumulator model

The LBA model is conceptually simple and yet can provide rich descriptions of behavior, rivaling those of the extended DDM (Brown and Heathcote, 2008; Donkin et al., 2011). Evidence *x*_*i*_(*t*) for each of two or more choices accumulates *linearly* and *ballistically* in time *t* from *x*_*i*_(0) = *x*_*i*0_* toward a threshold *z* at a drift rate μ^*^_i_:

(5) $$x_{i}(t)=x_{i0}^{*}+\mu_{i}^{*}t,i=1,2,\ldots ,N.$$

As in the extended DDM, parameters may vary from trial to trial. Here μ^*^_i_ is selected from 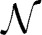 [μ_*i*_, *s*^2^] and x^*^_i0_ from 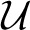
[0, *A*] on each trial. The parameter *A* > 0 defines the maximum value that any starting point x^*^_i0_ may assume. The accumulator *x*_*i*_(*t*) that is first to reach the threshold *z* is selected. In prior work, *A* has been restricted to lie below *z*, i.e., *A* ≤ *z* (Brown and Heathcote, 2008; Donkin et al., 2011). A non-decision time *T*_0_ is also included. While drift rates generally differ for each accumulator (μ_*i*_ ≠ μ_*j*_), the remaining parameters *A*, *z*, *s*, *T*_0_ are common to all accumulators.

Closed form expressions describing the LBA model's behavior were derived in Brown and Heathcote (2008). The cumulative distribution function (CDF), *F*_*i*_(*t*), and the probability density function (PDF), *f*_*i*_(*t*), can be written in terms of the LBA parameters for individual accumulators:

(6) $$\begin{array}{l} F_{i}(t)=1+\frac{z-A-t\mu_{i}}{A}\Phi \text{​}\left(\frac{z-A-t\mu_{i}}{ts}\right)\\ \;-\frac{z-t\mu_{i}}{A}\Phi \text{​}\left(\frac{z-t\mu_{i}}{ts}\right)+\frac{ts}{A}\phi \text{​}\left(\frac{z-A-t\mu_{i}}{ts}\right)\\ \;-\frac{ts}{A}\phi \text{​}\left(\frac{z-t\mu_{i}}{ts}\right), \end{array}$$

(7) $$\begin{array}{l} f_{i}(t)=\frac{1}{A}[-\mu_{i}\Phi \text{​}\left(\frac{z-A-t\mu_{i}}{ts}\right)+s\phi \text{​}\left(\frac{z-A-t\mu_{i}}{ts}\right)\\ \;+\mu_{i}\Phi \text{​}\left(\frac{z-t\mu_{i}}{ts}\right)-s\phi \text{​}\left(\frac{z-t\mu_{i}}{ts}\right)], \end{array}$$

where Φ(·) is the CDF and ϕ(·) the PDF for the normal distribution with zero mean and unit variance. See Brown and Heathcote (2008, Supplementary Material) for the derivations of Equations (6, 7).

To determine mean first passage times for competing accumulations, we use the *defective PDF*, denoted PDF_i_(*t*); unlike the standard PDF, the defective PDF generally integrates to a value between 0 and 1. PDF_i_(*t*) describes the likelihood that accumulator *x*_*i*_(*t*) reaches the threshold *provided that no other accumulator has already done so*:

(8) $${\text{PDF}}_{i}(t)=f_{i}(t)\prod_{j\neq i}\left(1-F_{j}(t)\right).$$

Because drift rates μ^*^_i_ are selected from a normal distribution, in some cases all μ^*^_i_'s are negative. When this happens, the model produces an infinite decision time, and no response is given. Thus to compare LBA responses to those predicted by the two DDMs, which are finite on every trial, we consider only simulated LBA trials that yield a finite response time, i.e., that have at least one accumulator with a positive drift rate on that trial. To do so we modify the expressions above by a normalization factor $\alpha (\mu_{1},\ldots ,\mu_{N},s)=1-\prod_{i\;=\;1}^{N}\Phi \left(-\frac{\mu_{i}}{s}\right)$, which is the probability that no accumulator reaches threshold. This follows since $\Phi \left(-\frac{\mu_{i}}{s}\right)$ is the probability that the *i*th accumulator has μ^*^_i_ < 0. The normalized defective probability density functions *p*_*i*_(*t*) are given in Brown and Heathcote (2008) as

(9) $$p_{i}(t)=\frac{{\text{PDF}}_{i}(t)}{\alpha (\mu_{1},\ldots ,\mu_{N},s)}.$$

For a two choice task, we therefore have

(10) $$p_{1}(t)=\frac{{\text{PDF}}_{1}(t)}{1-\Phi \left(-\frac{\mu_{1}}{s}\right)\Phi \left(-\frac{\mu_{2}}{s}\right)},$$

(11) $$p_{2}(t)=\frac{{\text{PDF}}_{2}(t)}{1-\Phi \left(-\frac{\mu_{1}}{s}\right)\Phi \left(-\frac{\mu_{2}}{s}\right)},$$

with ⨛^∞^_0_ (*p*_1_(*t*) + *p*_2_(*t*)) *dt* = 1. The expressions for DT and ER are

(12) $$\text{DT}=\int_{0}^{\infty }t(p_{1}(t)+p_{2}(t))dt,$$

(13) $$\text{ER}=\int_{0}^{\infty }p_{2}(t)dt.$$

Following the convention adopted for the DDM, we shall assume that μ_1_ ≥ μ_2_, so that *p*_1_(*t*) and *p*_2_(*t*) represent correct and incorrect responses, respectively, and the corresponding DTs may be written as

(14) $${\text{DT}}_{\text{correct}}=\frac{1}{1-\text{ER}}\int_{0}^{\infty }tp_{1}(t)dt,$$

(15) $${\text{DT}}_{\text{error}}=\frac{1}{\text{ER}}\int_{0}^{\infty }tp_{2}(t)dt.$$

We also normalize the sum of the mean drift rates: μ_1_ + μ_2_ = 1. For the LBA described in the literature (Brown and Heathcote, 2008; Donkin et al., 2011), the threshold must not fall within the range of starting points, i.e., we must have *z* ≥ *A*. The LBA, unlike the two DDMs, therefore almost always predicts non-zero DTs. Implications of this in determining an optimal speed-accuracy tradeoff are discussed in the next section.

### 2.2. Optimal performances

As in Bogacz et al. (2006), we define optimal performance as a strategy that maximizes the Reward Rate (RR):

(16) $$\text{RR}=\frac{1-\text{ER}}{\text{DT}+T_{0}+\text{RSI}},$$

where RSI denotes the response to stimulus interval (see section 2.3 below). To assess performance, we seek a relationship between the behavioral measures ER and DT that yields the maximum RR for a given decision making model. This Optimal Performance Curve (OPC) (Bogacz et al., 2006) relates normalized DT to ER, where the former is defined as $\frac{\text{DT}}{{\text{D}}_{\text{tot}}}$ with D_tot_ = *T*_0_ + RSI. We now describe OPCs for the DDM, extended DDM, and LBA.

#### 2.2.1. Optimal performance under the pure DDM is described by a unique curve

**Figure 2:**
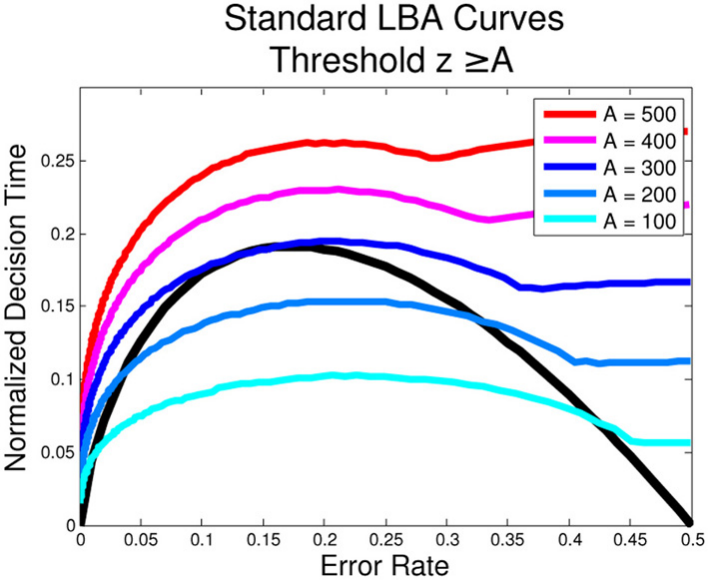
**Optimal Performance Curves (OPCs) for the LBA are not unique**. The following parameters for the standard LBA were used in the simulation: *s* = 0.32, *T*_0_ = 226 ms, RSI = 1000 ms, μ_1_ + μ_2_ = 1. The unique OPC of the pure DDM is shown in black.

The pure DDM has a unique, parameter-free OPC, defined by

(17) $$\frac{\text{DT}}{{\text{D}}_{\text{tot}}}={\left(\frac{1}{\text{ER}\log\frac{1-\text{ER}}{\text{ER}}}+\frac{1}{1-2\text{ER}}\right)}^{-1},$$

which is derived by finding the threshold that maximizes RR for a given task difficulty (Bogacz et al., 2006). This function is shown in solid black in Figure 2 below. Its general shape can be intuitively explained by noting that for very noisy stimuli, prolonged evidence accumulation cannot improve much over random choices, so at the righthand end optimal thresholds approach zero, DT → 0 and ER → 0.5. Alternatively, very easy tasks require little accumulation to achieve high accuracy, so DTs are also small at the left, but ER → 0. For each intermediate difficulty level and a given D_tot_ = *T*_0_ + RSI, there is a unique optimal threshold with associated DT and ER between 0 and 0.5 that maximizes RR, thus defining the curve. All other thresholds, associated with faster or slower RTs, yield smaller net rewards at that task condition. Going from right to left, RRs rise as difficulty decreases, but it is important to recognize that any point on the OPC corresponds to maximum RR for a specific task condition. See Bogacz et al. (2006) and Zacksenhouse et al. (2010) for further discussion and illustrations of the OPC.

#### 2.2.2. Optimal performance under the extended DDM is not uniquely defined

The extended DDM has families of OPCs rather than a unique OPC, as in the pure DDM. In the extended DDM, variability in starting points precludes the possibility of trials with a DT = 0. However, for low values of variance parameters in the extended DDM, the OPCs for the extended DDM approach the OPC for the pure DDM. For a sample OPC for the extended DDM see (Bogacz et al., 2006, Figure 14). To compute such curves, all parameters except drift rate and threshold are fixed. Then the threshold which optimizes RR is computed for each drift rate and used to determine ER and normalized DT. Further details can be found in Bogacz et al. (2006).

#### 2.2.3. Optimal performance under the LBA is not uniquely defined

The LBA expressions of Equations (6–11) are complicated, and simple analytical expressions of their OPC families do not seem possible. Instead we approximate them numerically. To do this, we fix *T*_0_, RSI, *s*, set μ_1_ + μ_2_ = 1 and choose *A*. We then calculate ER and DT for each μ_1_ ∈ [0.5, 1]. From these we estimate the optimal *z* and find the corresponding ER and DT, producing a point on the OPC for the selected *A* value. We find that a different OPC is generated for each value of *A*, as shown in Figure 2, i.e., there is no unique OPC for the LBA.

This is consistent with the observation that, for μ_1_ = μ_2_ = 0.5 (equal evidence for both options), different choices of *A* will affect the DT but not the ER. This is because the expected accuracy will be exactly 0.5 and no greater accuracy may be realized or information accumulated over time. It follows that the optimal solution is the lowest possible threshold and therefore the shortest possible DT.

For the pure DDM and the extended DDM with zero starting point variance (*s*_*x*_ = 0), the threshold parameter, *z*, can go to 0, and likewise the DT. However, in the LBA, the threshold must lie at or above the top of the range of starting points, i.e., *z* ≥ *A*. Since this is a key source of variability, in general *A* > 0. Moreover, the lowest (and optimal) threshold for μ_1_ = μ_2_ = μ is therefore *z* = *A*, which gives DT > 0. The OPC curve plotting $\frac{\text{DT}}{{\text{DT}}_{\text{tot}}}$ varies with the value of *A* as shown in Figure 2; the smooth portions of each curve correspond to *z* > *A* (on the left) and *z* = *A* (on the right). That is, when the task is difficult and the ER is close to 0.5, the threshold, *z*, is as small as it can be. Moreover, unlike the OPC for the DDM, the OPCs for the LBA terminate at ER = 0.5 with finite normalized DTs. As *A* → 0, the normalized DTs approach 0 for all ERs.

#### 2.2.4. An adapted LBA allows fast responses at high error rates

The above analysis prompts us to define an *adapted version of the LBA*, in which thresholds can take values in the range of starting points, i.e., *z* ≥ *A* is relaxed to *z* ≥ 0. If the starting point in one or both accumulators is greater than *z*, then DT = 0 and the accumulator with the higher starting point is selected for that trial. The mean error rate and decision time, ER_a_ and DT_a_, for the adapted DT with *z* < *A* are defined accordingly:

(18) $${\text{ER}}_{\text{a}}(A,z,\mu_{1},\mu_{2},s)=\frac{1}{2}\cdot \frac{A-z}{A}+\frac{z}{A}\cdot \text{ER}(z,z,\mu_{1},\mu_{2},s),$$

(19) $${\text{DT}}_{\text{a}}(A,z,\mu_{1},\mu_{2},s)=\frac{z}{A}\cdot \text{DT}(z,z,\mu_{1},\mu_{2},s),$$

where ER and DT are calculated as in Equations (12–15) for the standard LBA.

Instantaneous decisions occurring for starting points above *z* can be seen as representing a prior resolution to respond as fast as possible, as is optimal for entirely noisy (zero-coherence) stimuli, see the OPC of Figure 2. In this case RT = *T*_0_. Hence the adapted LBA can produce bimodal RT distributions, with a delta function at *T*_0_ for trials with starting points above *z* and a second peak at longer RTs due to those starting below *z*.

Numerically-derived OPCs for the adapted LBA are also non-unique. As stimuli become less discriminable and ERs approach 0.5, the best values of *z* are those that minimize DT. Hence *z*_*opt*_ → 0 as μ_1_ → μ_2_, leading to many rapid responses. For μ_1_ > > μ_2_, we also find *z*_*opt*_ → 0 and DT → 0, but with ER → 0 due to fast drift toward correct choices. As *A* varies this produces a family of OPCs with portions on the left similar to those of Figure 2, but approaching DT = 0 as ER → 0.5. Also, as *A* → 0, *z*_*opt*_ → 0 for μ_1_ ≠ μ_2_, as for the standard LBA.

#### 2.2.5. Noise scales differently in the pure DDM and standard LBA

Poorly discriminable stimuli correspond to low signal-to-noise ratios μ / σ in the pure DDM, and may also correspond to variability in drift rates and starting points in the extended DDM. The LBA has trial-to-trial variability (“noise”) in drift rates and starting points but lacks additive noise in individual trials. We now compare noise scaling in the two models in the case of equal mean evidence for both alternatives, represented as μ_1_ = μ_2_ in the LBA model and as μ = 0 in the DDM. We show that the DDM and LBA behave differently as noise increases.

We first consider approximations of DT and ER about the point $\frac{1}{\sigma^{2}}=0$ (analogous to μ = 0) for the DDM. Taking μ and *z* fixed and strictly positive, and expanding the expressions (2, 3) with respect to the small parameter $\frac{1}{\sigma^{2}}$ in Taylor series, we obtain

(20) $$\text{DT}\left(\frac{1}{\sigma^{2}}\right)=z^{2}\left(\frac{1}{\sigma^{2}}\right)-\frac{\mu^{2}z^{4}}{3}\left(\frac{1}{\sigma^{6}}\right)+O\left(\frac{\mu^{4}z^{6}}{\sigma^{10}}\right)\text{ and}$$

(21) $$\text{ER}\left(\frac{1}{\sigma^{2}}\right)=\frac{1}{2}-\frac{z\mu }{2}\left(\frac{1}{\sigma^{2}}\right)+\frac{z^{3}\mu^{3}}{6}\left(\frac{1}{\sigma^{6}}\right)+O\left(\frac{z^{5}\mu^{5}}{\sigma^{10}}\right)\text{​​}.\;$$

Here ER is *O* (1), and DT is $O\left(\frac{1}{\sigma^{2}}\right)$. Thus, ER scales differently with high noise σ as compared to DT. In particular, as σ → ∞, ER→ 0.5 and DT→ 0. Intuitively, large noise pushes the accumulation process rapidly toward one of the boundaries.

We now consider scaling of ER and DT with noise in the standard LBA, and show that large noise generally leads to non-zero DTs and always leads to non-zero mean DTs, given non-zero thresholds. For non-discriminable stimuli $\mu_{1}=\mu_{2}=\frac{1}{2}$ the LBA has two sources of noise or variability: in drift rates, *s*, and in starting points, *A*. In all cases, since μ_1_ = μ_2_ and drift is the only source of bias, ER = 0.5. We note that the mean DT can be 0 if and only if *A* = 0, due to the constraint that *z* ≥ *A*, so that *A* ≠ 0 implies a non-zero distance to accumulate to threshold. To see this, first suppose that *s* = 0 and then allow *s* to increase, producing a distribution of drift rates centered around $\mu_{i}=\frac{1}{2}$. In fact for *s* = 0 the RT distribution is

(22) $$f(t)=\{\begin{array}{l} 0,\;t<\frac{z-A}{\mu }\\ \frac{2\mu Z}{A^{2}}\left(1-\frac{\mu t}{z}\right),t\in \left[\frac{z-A}{\mu },\frac{z}{\mu }\right]\\ 0,\;t>\frac{z}{\mu } \end{array},$$

with mean DT = (3*z* − 2*A*) / 3 μ, and this value will change continuously with *s*. It follows that for μ_1_ = μ_2_ and the minimum z^*^ = *A*, DT increases with *A*. This behavior for μ_1_ = μ_2_ and high *A* is quite different from that of the optimized pure DDM, in which large noise implies that mean DT → 0.

### 2.3. Reward maximization experiment

In order to compare model fits, we reanalyze human behavioral data from a free response motion discrimination task previously presented in Balci et al. (2011). Participants (*n* = 17, 6 male) were asked to discriminate the direction of displays of moving dots on a computer screen and instructed to maximize their rewards. Task difficulty was adjusted via motion coherence determined by the fraction of dots moving leftward or rightward while the rest moved randomly.

Stimuli were viewed at ≈ 60 cm from the CRT monitor. The participant indicated motion direction by pressing a key on a standard keyboard: leftward with the “Z” key and rightward with the “M” key. Leftward and rightward stimuli were presented with equal probabilities. Premature responses, either anticipatory or with RTs of less than 100 ms, were penalized with a buzzing sound and a 4 s timeout period. When participants did not respond prematurely, RSIs were selected from a truncated exponential distribution with mean of 1 s. Experiments were conducted at a Macintosh computer, using the Psychophysics Toolbox (Brainard, 1997).

Each participant completed at least 13 daily sessions of 60 min total duration. The first four of these sessions involved training and practice without monetary reward. In each of the remaining sessions, participants completed five blocks with motion stimuli presented at a different coherence in each block (0, 4, 8, 16, and 32%, randomized across participants); participants earned $0.02 for each correct response. Performance improved markedly over the first 5 sessions and for certain participants continued to improve until session 9. Here we only use data from sessions 10–13.

After completing the motion discrimination task, participants performed an interval timing task and a signal detection task. The signal detection task was the same as above, except that participants were instructed to respond merely to motion onset, regardless of direction. In one block they were instructed to press the “M” key, and in the other, the “Z” key, again receiving $0.02 for each correct (non-anticipatory) response. The signal detection data was used to compute non-decision times as described in the following section. Interval timing data is not used here, so we do not describe that task. For more details, see Balci et al. (2011).

### 2.4. Data fitting procedures

Fits were performed to participant data for the two DDMs and the LBA using published toolboxes for the models in Matlab (Tuerlinckx, 2004) and R (Donkin et al., 2009), respectively. Fits to the LBA model required some modifications to the standard LBA code, as outlined in Donkin et al. (2009).

Data were separated by difficulty and fits were computed for individual participants over all five difficulty conditions. Multiple fits were performed for each condition and participant, first varying only drift rate and caution (threshold) with difficulty level for the pure DDM, and then also varying the range of starting points, while the remaining parameters were held constant. The DDMs and LBA were fit separately to RT distributions for correct and error trials in each condition using five quantiles (10%, 30%, 50%, 70%, 90%). The same data and partitioning were used for both model fit toolboxes. The toolboxes fit non-decision times *T*_0_, so that mean RTs and DTs can be computed for all models.

**Figure 3:**
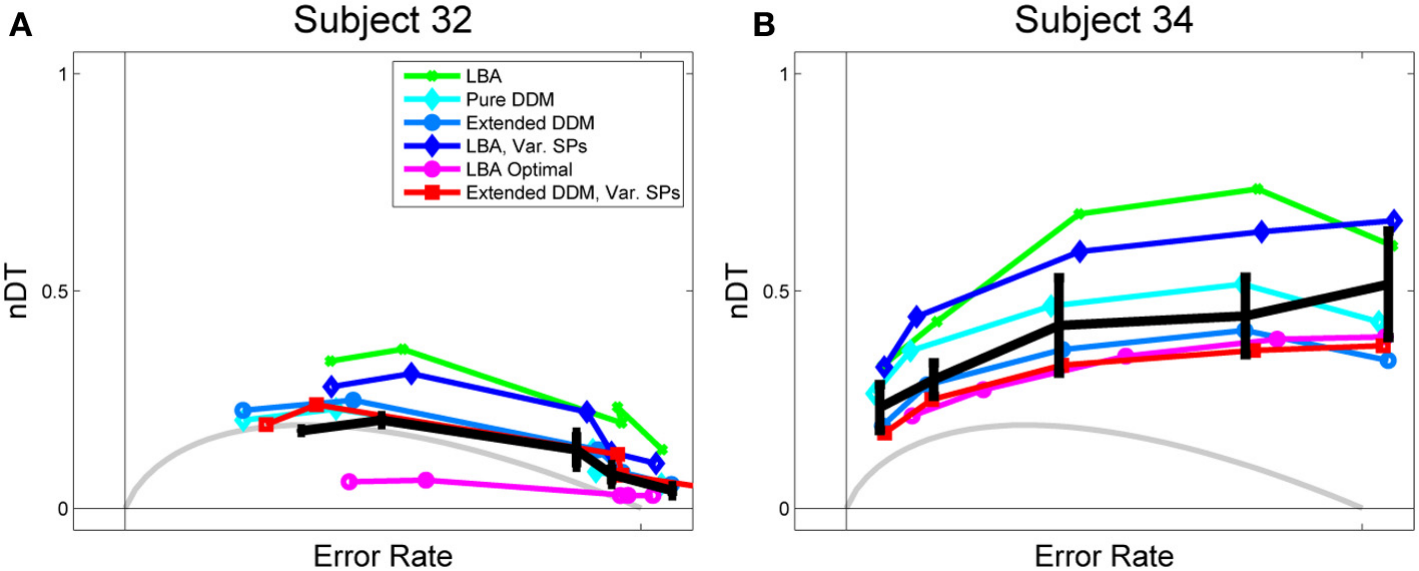
**Data and fits to Rmax behavior for (A) a high performing and (B) a low performing participant**. Empirical normalized DTs, estimated from mean RT data for the main task and RTs from a signal detection task, are shown in black with standard error bars. OPC for the pure DDM is shown in gray, and OPCs for the LBA, computed as described in section 2.2.3, are shown in purple open circles. LBA fits, shown in green, overestimate normalized DTs for both subjects. The OPC for the LBA, however, lies significantly below the data for Subject 32, because this data requires a small starting point range *A* to get low DTs in difficult tasks, and a low *A* yields low DTs throughout.

**Figure 4:**
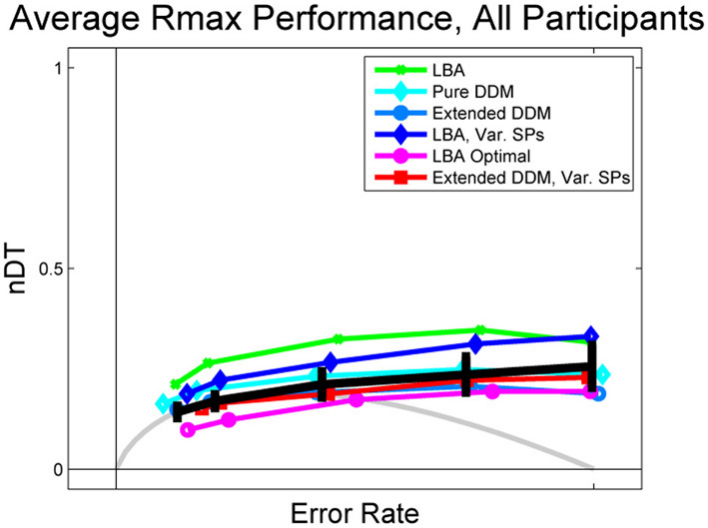
**Data and fits to Rmax behavior averaged over all participants, with comparison to OPCs for pure DDM (gray) and LBA (purple open circles)**. Data is shown in black, with error bars being the mean of standard errors for each participant within the given ER bin. LBA fits (green dots and dark blue diamonds) overestimate mean normalized DTs, but the OPC for the LBA underestimates them.

Empirical non-decision times were also estimated for each participant from their mean RTs for the fastest 25% of signal detection trials, as in Balci (Personal Communication, 2011) and Balci et al. (2011). These non-decision times were only used in computing normalized mean DTs for the human data shown below in Figures 3, 4 and Supplementary Figure S4; normalized mean DTs for the models were derived from the model fit toolboxes.

Both the DMAT (DDM) and LBA toolboxes allow the user to constrain some parameters to constant values while others are allowed to vary across conditions. The DMAT toolbox does this by using a combination of a system of matrix equations similar to those in general linear models, coupled with post-processing to remove outliers (Vandekerckhove and Tuerlinckx, 2007, 2008). The LBA toolbox uses the quantile maximum probability estimator method (Heathcote et al., 2004) to estimate PDFs for correct and error trials, which are then used to select model parameters.

The qualities of the resulting model fits were then assessed by comparing their predicted mean RTs and ERs with data for each condition and participant, using the Akaike, Corrected Akaike, and Bayesian Information Criteria (AIC, AIC_c_, and BIC) (Akaike, 1974, 1980, 1981). Finally, in addition to each participant's actual performance, a theoretically optimal performance for each difficulty condition was estimated by allowing the caution parameter to vary freely while holding all other parameters fixed at that participant's fitted values. The optimal value of caution is defined as that yielding the highest possible reward rate, given constant (fitted) values for the remaining parameters.

## 3. Results

To compare the properties of the DDM and LBA in fitting Rmax task data, we fitted the following models to data for each participant:

- A pure DDM, in which thresholds *z* and drift rates μ vary among coherence conditions (13 parameters: *x*_0_, σ^2^, *T*_0_, 5 *z*'s and 5 μ's).
- An extended DDM, in which thresholds *z* and mean drift rates μ^*^ vary among coherence conditions (16 parameters: *x*_0_, σ^2^, *T*_0_, 5 *z*'s, 5 μ's and *s*_*x*_, *s*_*T*_0__, *s*_μ_).
- A second extended DDM, in which thresholds *z*, mean drift rates μ^*^ and ranges *s*_*x*_ of starting points (SPs) vary among coherence conditions (20 parameters: *x*_0_, σ^2^, *T*_0_, 5 *z*'s, 5 μ's, 5 *s*_*x*_'s, *s*_*T*__0_ and *s*_μ_).
- A LBA model, in which thresholds *z* and mean drift rates μ_*i*_ vary among coherence conditions (13 parameters, with μ_1_ + μ_2_ = 1: *A*, *s*, *T*_0_, 5 *z*'s and 5μ_1_'s).
- A second LBA model, in which thresholds *z*, mean drift rates μ_*i*_ and ranges *A* of starting points (SPs) vary among coherence conditions (17 parameters, with μ_1_ + μ_2_ = 1: 5 *A*'s, *s*, *T*_0_, 5 *z*'s and 5 μ_1_'s).

Note that, for increased flexibility, σ is not set to 1.

AIC, AIC_c_, and BIC scores for each participant and model were computed based on mean RT and ER data and model predictions. These were then averaged over all participants and conditions to determine mean scores for each model. All three metrics reward goodness of fit while penalizing extra parameters; lower scores are desirable and negative values are possible (Akaike, 1974, 1980, 1981). The scores, along with mean values of the correlation coefficient *R*^2^, are summarized in Table 1. Figures S1 and S2 in the Supplementary Material show individual fits to RT and ER data. According to AIC, AIC_c_, and BIC, the pure DDM provides the best overall fit to mean RT and ER data, but fits for each participant and condition are quite good for all five models.

**Table 1:** **DDM and LBA model fit comparisons, with fit quality defined by the match to mean ER and RT data**.

**Model**	**Total parameters**	**AIC**	**AIC_c_**	**BIC**	***R*^2^**
Pure DDM	13	63.57	−1.43	93.50	0.98
Extended DDM	16	66.37	20.66	103.21	0.98
Extended DDM with var. SPs	20	66.76	30.40	112.81	0.99
LBA	13	80.44	15.44	110.37	0.95
LBA with var. SPs	17	66.18	23.68	105.32	0.99

Lower AIC, AIC_*c*_, and BIC scores indicate better fits. See text for discussion.

Lower AIC, AIC_c_, and BIC scores for the pure DDM are due to the fact that it has fewer parameters than all other models used here except the standard LBA, and the pure DDM predicts mean ER and RT data well. The extended DDM and LBA with variable starting points achieve slightly higher mean *R*^2^ values than the pure DDM, indicating better fits to mean behavior when additional parameters are included. Comparing the pure DDM fit to the extended DDMs over RT *distributions* using the DDM toolbox, the extended DDM (χ^2^ = 168.35, *p* < 0.05) and extended DDM with variable starting points (χ^2^ = 408.94, *p* < 0.001) yield superior deviance scores (Chernoff and Lehmann, 1954). However, fitting distributions is problematic because individual participant data sets separated by coherence condition are relatively small, and assessment of Rmax performance requires mean RTs and ERs.

Table 1 also shows that allowing the range of starting points to vary in the LBA yields better fits according to AIC and BIC but not AIC_c_, and that such variability in the extended DDM does not improve fits according to any of these metrics.

Figure 3 shows normalized DTs for representative high and low performing participants (Subjects 32 and 34) and Figure 4 shows the DTs averaged over all participants. Individual fits appear in Figure S4 of the Supplementary Material. The data is shown in solid black with error bars, and model fits are superimposed in curves of various colors with different markers: the LBA in green dots, the pure DDM in light blue with diamonds, the extended DDM in dark blue with circles, the LBA with varied starting point ranges in dark blue with diamonds, and the extended DDM with varied starting point ranges in red with squares. The OPC for the LBA is indicated in purple with circles, and the OPC for the DDM in light gray.

Figure 3 illustrates a key difference between high and low performing participants. For the former (Figure 3A), the fits all trend downward and mean normalized DTs decrease as ERs increase, as they do for the OPC for the DDM. For the latter (Figure 3B), this trend is reversed and all fits diverge from the OPC for the DDM. The pure and extended DDM fits lie close to the data in both cases. The OPC for the LBA generally predicts the smallest normalized DTs, but it lies far below the data at intermediate ERs for the high performing participant.

As with individual subjects who do not perform at the highest level (Figure 3B), the average behavior shown in Figure 4 diverges from the OPC for the pure DDM as ERs increase. The difference between the empirical normalized mean DTs of Figure 4 and the OPC for the pure DDM is a good predictor of overall RR for each of the coherence conditions (*R*^2^ = 0.53, *p* < 0.001).

The LBA models and the pure DDM overestimate normalized DTs and the extended DDMs slightly underestimate them. This is due in part to some subjectivity in the estimation of non-decision time *T*_0_. For example, the LBA tends to fit smaller *T*_0_ values than do the DDMs, and thus the LBA yields larger DTs (Donkin et al., 2011). However, while the LBA fits lie above the data curve, the OPC for the LBA lies below it, especially at intermediate ERs. This holds for many high performing participants (see Figure 3A and Supplementary Figure S4). For such individuals the range of starting points is small (Supplementary Figure S6), so that thresholds can be small without a major sacrifice in accuracy. We investigate how starting point ranges depend on coherence below (Figure 6A).

To better understand differences among the five models, we next consider mean parameter fits for the caution parameter, i.e., threshold, *z*. For each participant, coherence condition, and DDM fit, we calculated a threshold, and then averaged over individual threshold values for a given model and coherence. Thresholds for individual participants and coherence levels appear in Figure S5 of the Supplementary Material.

**Figure 5:**
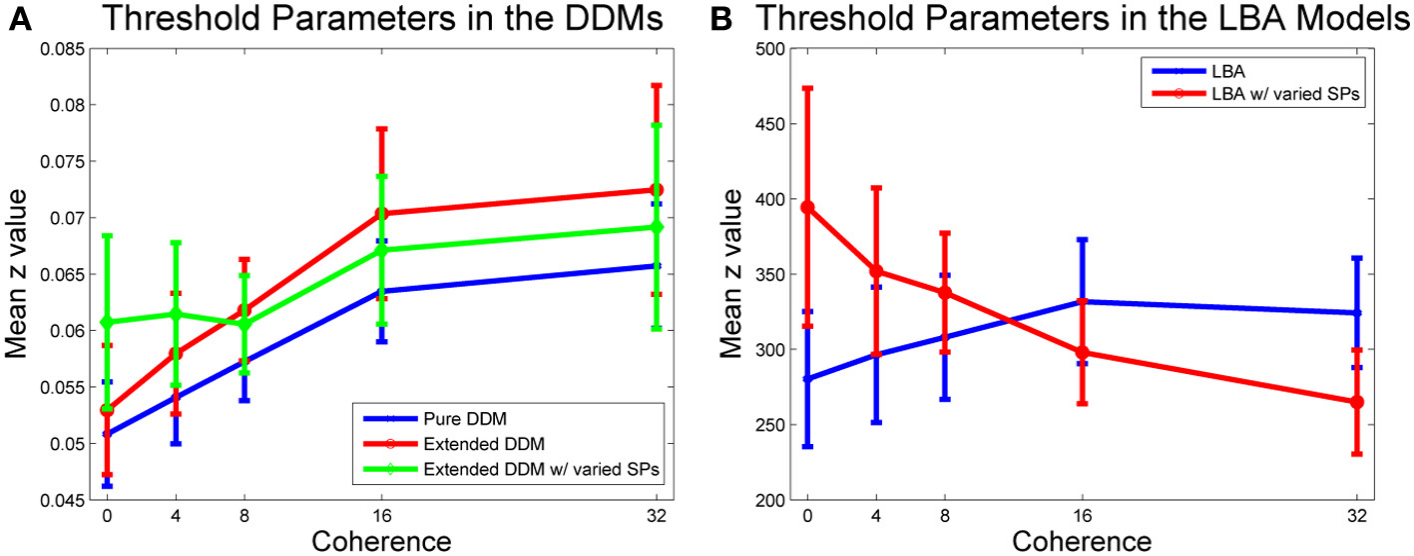
**Mean threshold values versus coherence**. **(A)** Caution increases with coherence in all DDM fits. Pure (blue) and first extended DDM (red) fits do not differ significantly, but difficulty condition is significant [*F*_(4, 64)_ = 3.91, *p* < 0.01, η^2^ = 0.16]. Allowing the starting point range to vary in the second extended DDM (green) does not improve fits by AIC/BIC, but compared with the other two DDM fits, main effects of model [*F*_(2, 32)_ = 3.99, *p* < 0.05, η^2^ = 0.02] and interaction of model type and difficulty condition [*F*_(8, 128)_ = 2.06, *p* < 0.01, η^2^ = 0.01] are significant. **(B)** Caution increases with coherence in the first LBA fit, and decreases with coherence in the second LBA fit, with variable starting point range. A within-groups ANOVA shows that main effect of model type [*F*_(1, 16)_ = 5.62, *p* < 0.05, η^2^ = 0.01] and interaction of model type and difficulty condition [*F*_(4, 64)_ = 4.29, *p* < 0.01, η^2^ = 0.08] are both significant. Bars indicate standard errors for *n* = 17 subjects.

Figure 5A shows that mean threshold values increase with coherence for all three DDMs. Allowing starting point ranges to vary with coherence in the extended DDM increased thresholds at the two lowest coherence levels, and this variation with coherence over all thresholds is significant in the extended DDM [*F*_(4, 64)_ = 73.72, *p* < 0.01, η^2^ = 0.82]. Extended DDM fits to the same data set in Balci et al. (2011) suggested approximately equal evidence for both variable *and* constant thresholds, because of differences in outlier treatment and in fitting algorithm options.

Figure 5B illustrates a similar analysis for the two LBA model fits. The fit to the LBA model with starting point range fixed over coherences also indicates that caution increases with coherence, but allowing starting point ranges to vary with coherence reverses this trend. A within-groups ANOVA on parameter values for the LBA with and without variability in starting point ranges shows that the main effect of LBA model type [*F*_(1, 16)_ = 5.62, *p* < 0.05, η^2^ = 0.01] and the interaction of LBA model type and difficulty condition [*F*_(4, 64)_ = 4.29, *p* < 0.01, η^2^ = 0.08] are both significant.

**Figure 6:**
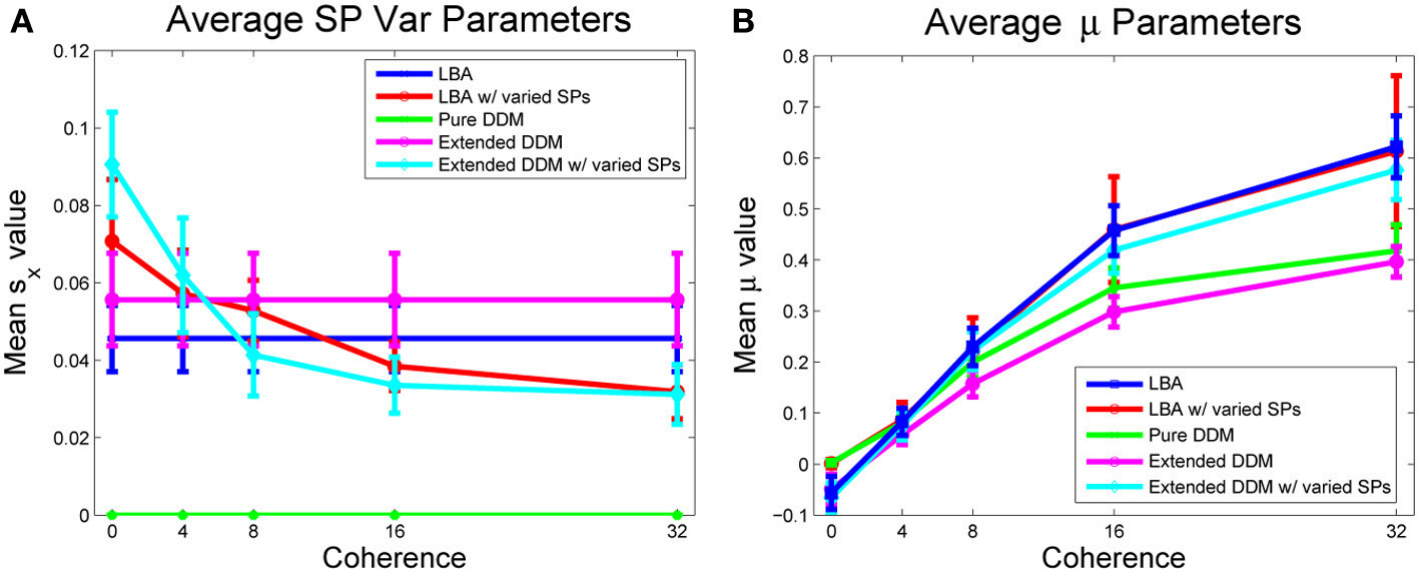
**(A)** Mean values of the range of starting points *s*_*x*_, *A*, averaged over participants, by model and coherence condition (the pure DDM does not allow starting point variability). **(B)** Mean values of drift rate μ, averaged over all participants. For LBA model fits, the relative evidence $\mu_{1}-\frac{1}{2}$ in favor of option 1 is shown. Bars indicate standard errors for *n* = 17 subjects.

We next consider the role of starting point variability. Mean values of starting point ranges, averaged across all participants, are shown in Figure 6A for all models. The two models in which variability is allowed exhibit similar monotonically decreasing starting point ranges as coherence increases. Analogous data for individual participants appears in Figure S6 of the Supplementary Material, illustrating substantial variability among participants.

Figure 6B compares the DDM and LBA estimates of mean drift rates. Here LBA drift values are reduced by subtracting $\frac{1}{2}$ from μ_1_, so that μ = 0 corresponds to zero coherence in both models, allowing direct comparisons. All models predict a monotonically increasing mean drift rate with increasing coherence. The effect of coherence on drift parameters is significant for all models with a large effect size [*F*_(4, 64)_ = 118.80, *p* < 0.001, η^2^ = 0.76]. Interaction of model and condition type is also significant, but effect size is modest [*F*_(16, 256)_ = 5.48, *p* < 0.001, η^2^ = 0.11]. The effect of model type on drift is also significant [*F*_(4, 64)_ = 7.79, *p* < 0.001, η^2^ = 0.10]. Drift rate estimates for individual participants appear in Figure S7 of the Supplementary Material, showing more uniformity than the starting point ranges of Supplementary Figure S6. Thus, estimates of task difficulty are in general agreement across all models.

## 4. Discussion

In this paper we compare accounts of behavior provided by fitting DDM and LBA models to behavioral data from a 2AFC Rmax task. Adjustments of thresholds, equivalent to caution, are known to be central to DDM descriptions of Rmax behavior. For example, participants may adjust their thresholds to best suit each difficulty condition (Bogacz et al., 2006, 2010; Balci et al., 2011), or pick a single threshold that works well, albeit suboptimally, across multiple difficulty levels (Balci et al., 2011).

We first showed that, while the optimal performance curve (OPC) for the pure DDM is unique and parameter free, OPCs for the LBA are non-unique (Figure 2), like those for the extended DDM. Moreover, for a given parameter set, optimal behavior in the LBA is at least partially determined by the range of starting points, *A*. If *A* > 0, fast responses at near signal detection speed are impossible because of thresholds *z* ≥ *A*, and if *A* ≈ 0, the quality of fits is limited. Lacking diffusive noise during trials, the LBA requires variable starting points as well as variable drift rates to produce a range of DTs and corresponding estimated RT. Allowing *A* to vary with task difficulty yields significantly better fits, and this parameter variability is consequently critical to the success of the LBA. We also proposed an adapted LBA, in which thresholds can lie below *A*, which we intend to analyze and fit to data in the future.

We then fitted five models to an Rmax data set: a pure DDM, an extended DDM, an extended DDM allowing starting point ranges that vary with task difficulty, a standard LBA, and an LBA that allows starting point ranges to vary with task difficulty. For consistency, we employed the LBA parameterization of Brown and Heathcote (2008) and Donkin et al. (2011) to parallel that of the extended DDM. We found that DDMs yielded somewhat better fits with a single starting point range. The AIC and BIC criteria indicated improved fits for the LBA with varying starting point ranges, although AIC_c_ did not (Table 1).

In all three DDMs and the LBA with a common starting point range, participants modestly increased caution with coherence. In contrast, allowing starting point ranges to vary in the LBA predicted that participants *decreased* caution with coherence (compare Figures 5A,B). However, starting point ranges decreased with coherence in both models that allowed variability (Figure 6A). The LBA models and the extended DDMs require thresholds to lie at or above starting point ranges, but the additional source of within-trial randomness in the DDM may allow smaller starting point ranges, and hence yield better fits for difficult stimuli. Thresholds can be arbitrarily low for the pure DDM, since it has a single starting point. All models agree that drift rates increase with coherence (Figure 6B).

Critically, then, the Rmax task reveals that the DDMs and the LBA with varying starting point ranges provide fundamentally different accounts of behavior. In the DDMs, increased participant caution accounts for changes in behavior as stimulus coherence increases from 0% to 32%. According to the LBA model with variable starting point range, participants instead decrease caution and simultaneously reduce their range of starting points as coherence increases. Consequently, mean accumulation distances can still decrease with coherence in the LBA, in spite of smaller starting points. The corresponding RT and ER data therefore remain comparable between LBA and DDM fits. However, interpretations of the role of caution in these fits are inconsistent, adding to earlier findings that LBA parameters may not correlate straightforwardly with those of the DDM (van Ravenzwaaij and Oberauer, 2009; Heathcote and Hayes, 2012).

Our results raise the broad question of model design and selection, and suggest several directions for future work. While good overall, neither the LBA nor DDM accounts of behavior are perfect. The pure DDM is analytically tractable and predicts a unique, parameter-free OPC against which Rmax task performances can be assessed (Bogacz et al., 2006), but it can fail to fit RT distributions, especially when correct and error trials are separated (Ratcliff and Smith, 2004). Additional freedom in extended DDMs with variable drift rates and starting points across trials allows good fits, but defies analytical description and produces multiparameter families of OPCs. The LBA, incorporating trial-to-trial variability but omitting diffusive noise within trials, is almost as simple and tractable as the pure DDM, but it yields families of OPCs in which the allowed range of starting points plays a central and apparently complex role. In contrast, shifting the unique starting point of the pure DDM makes clear predictions regarding biased stimuli or incentivized rewards (Feng et al., 2009; Simen et al., 2009; Rorie et al., 2010; Gao et al., 2011).

Future work might re-adjust our interpretation of LBA parameters. For example, one might assume that the starting point range is controlled in tandem with thresholds. Indeed, a recent study suggests that this range narrows with practice (Heathcote and Hayes, 2012). The adapted LBA introduced in section 2.2.4 may provide accounts of average behavior that are more consistent with those provided by DDM fits, and, as noted there, it can produce bimodal RT distributions such as those sometimes observed for low stimulus discriminability, e.g., Simen et al. (2009) and Balci et al. (2011). Additional numerical and theoretical analyses are also of interest. For example, following Teodorescu and Usher (2013) one could compare models such as the Leaky Competing Accumulator as well as optimal Bayesian accounts of behavior with LBAs and DDMs.

### Conflict of interest statement

The authors declare that the research was conducted in the absence of any commercial or financial relationships that could be construed as a potential conflict of interest.
