# Supplementary material for: A comparative study of drift diffusion and linear ballistic accumulator models in a reward maximization perceptual choice task
Source: Front Neurosci. 2014 Aug 5;8:148. doi: 10.3389/fnins.2014.00148 (PMC4122186; doi:10.3389/fnins.2014.00148)
Supplement: Supplementary file 1 [file Presentation1.PDF]

1. Supplementary Material

Here we provide additional details on individual participant fits.

Figures 7-13 show details of fits to individual participants for the five models, averaged over all trials in sessions 10-13, for each given coherence. Specifically, Figures 7 and 8 show RTs and ERs versus coherence for the experimental data, and as predicted by fits of the five models. Figure 10 shows the mean normalized DTs versus ERs in comparison with the unique OPC for the pure DDM. Finally, Figures 9, 11, and 12 show reward rates (RR), thresholds (z), and starting point variability (s_x, or A) versus coherence for the model fits.

Participants are presented from left to right and top to bottom in each figure, based on closeness of behavior to that predicted by the OPC for the pure DDM. Closeness is measured by mean square differences between mean normalized DTs and mean normalized DTs on the OPC computed for each individual. The participant with the smallest score, Subject 39, appears in the upper left corner of each figure, and the participant with the highest score, Subject 17, appears in the lower right corner.

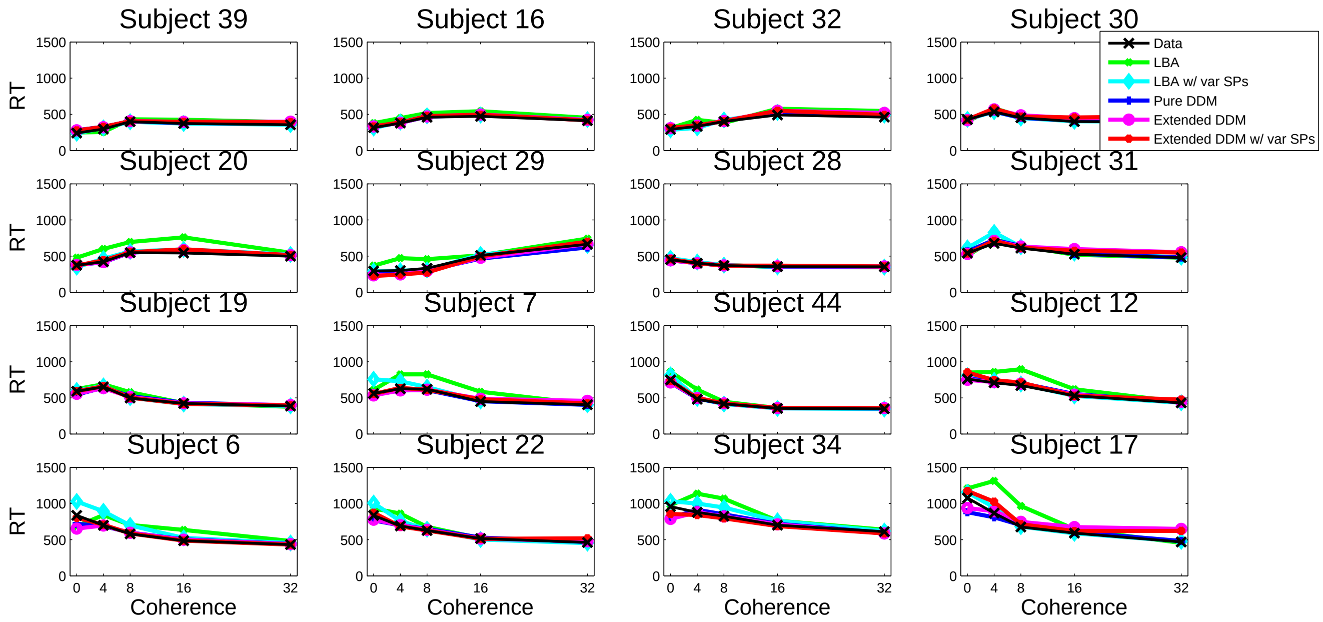

FIGURE 7. Comparison of data and fits to RT, in msec, by participant and closeness to OPC for the DDM. Several participants have relatively long RTs on the hardest tasks (0 and 4% coherence), for which optimal behavior requires responses at or near signal detection speed.

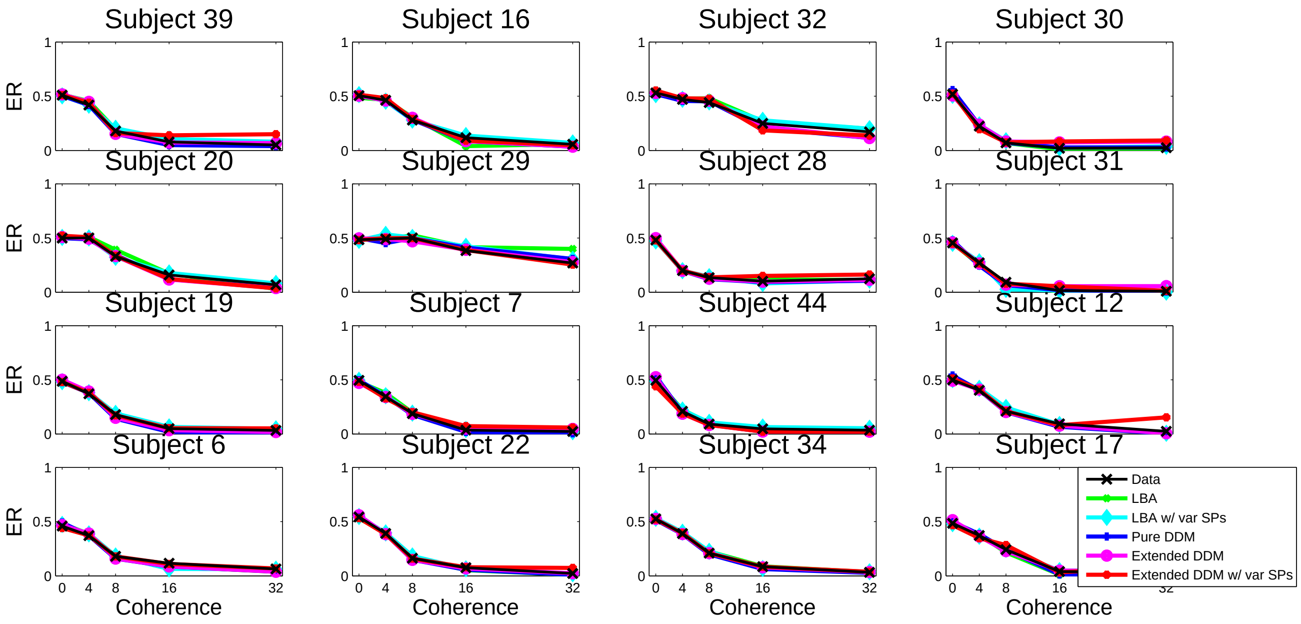

FIGURE 8. Comparison of data and model fits to ER, by participant and closeness to OPC for the DDM. Several participants (e.g. Subjects 22, 30, and 32) performed at worse than chance accuracy in the 0% coherence condition, and all models captured this behavior.

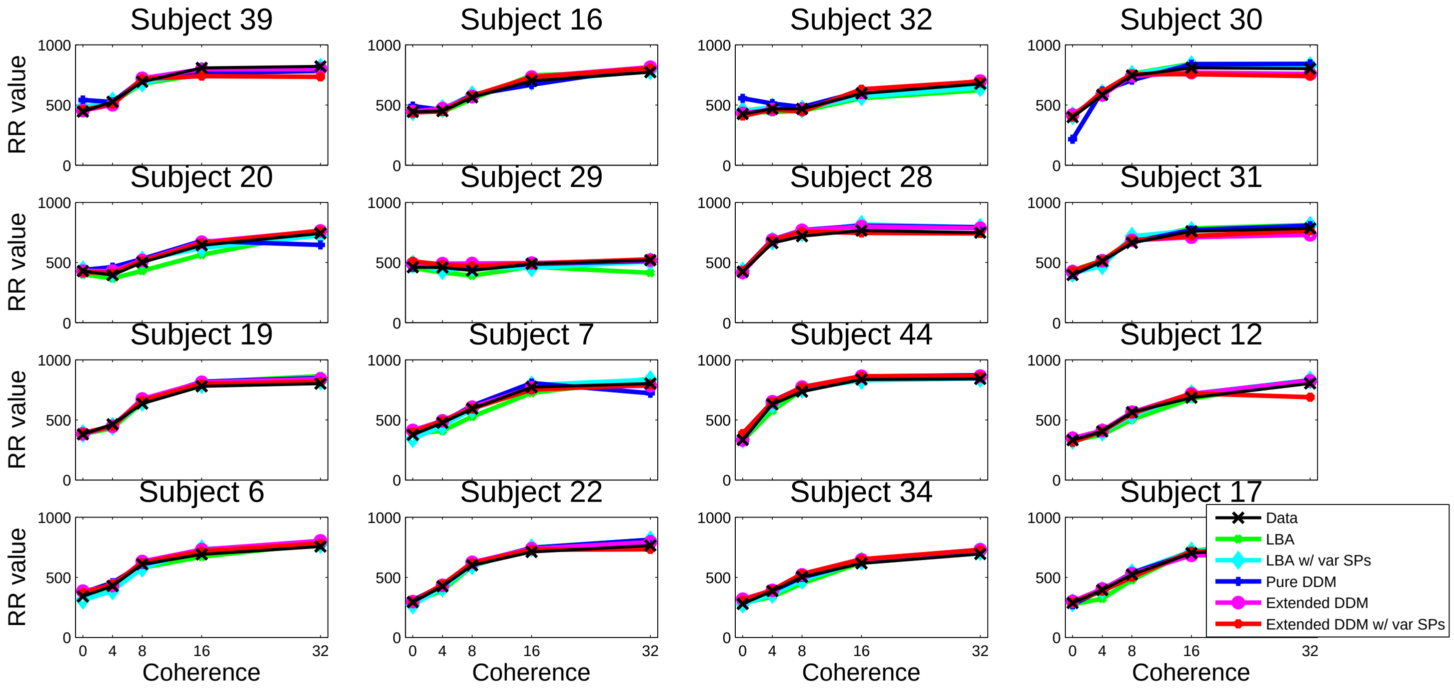

FIGURE 9. Comparison of data and model fits to RR (total rewards earned in Sessions 10-13) by participant and closeness to OPC for the DDM. Subject 29 had an unusual near-flat response curve.

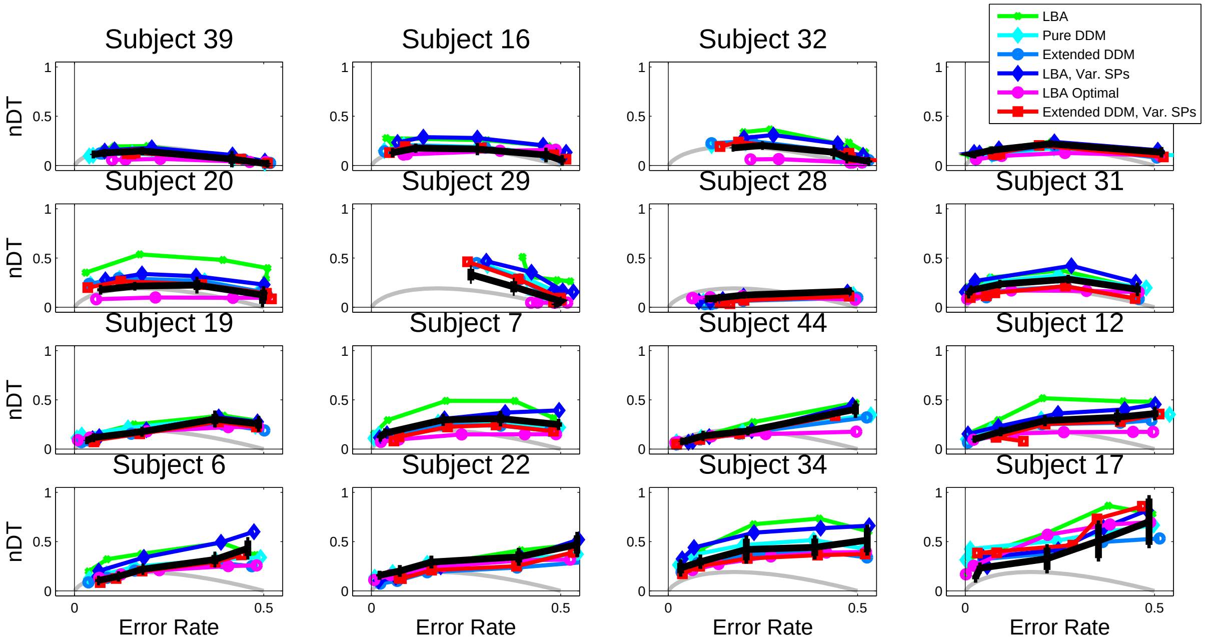

FIGURE 10. Comparison of data and model fits to mean normalized DT and ER data by participant and closeness to OPC for the DDM. Data is shown in black, with error bars for each ER bin; OPC for pure DDM shown in gray. Some differences among LBA and DDM predictions are due to differences among model estimates of nondecision time.

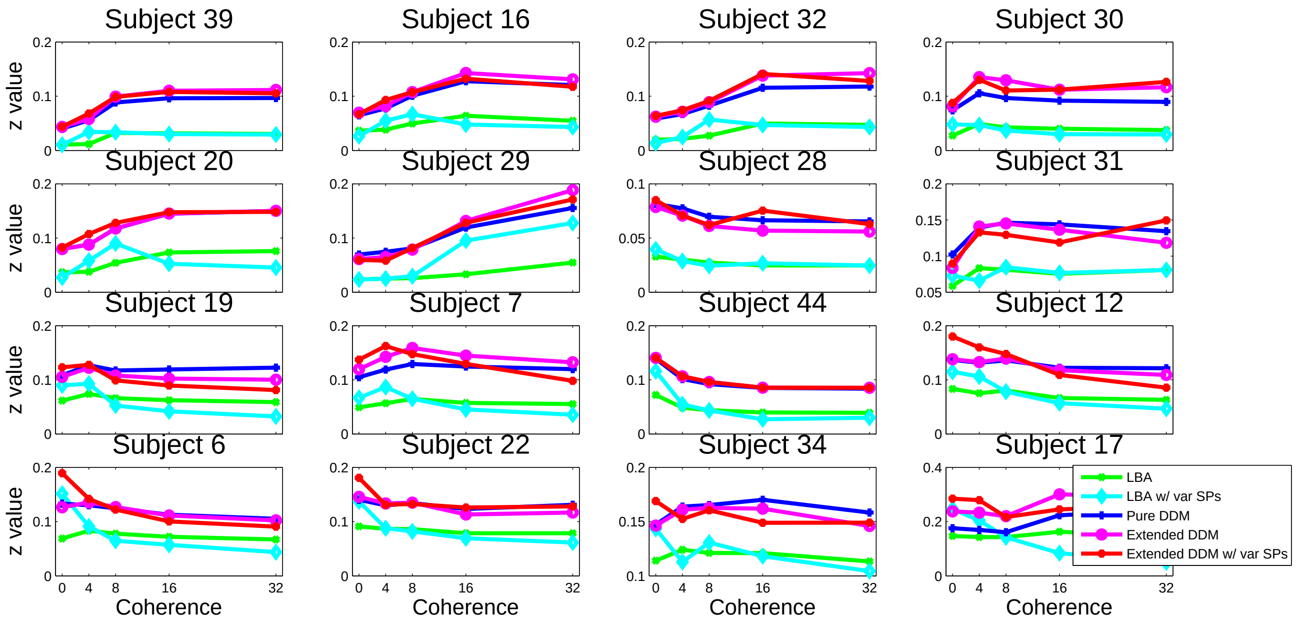

FIGURE 11. Comparison of caution or threshold parameter model fits, by participant and closeness to OPC for the DDM. LBA values z have been scaled down by a factor of 5000, to fit on the same axis.

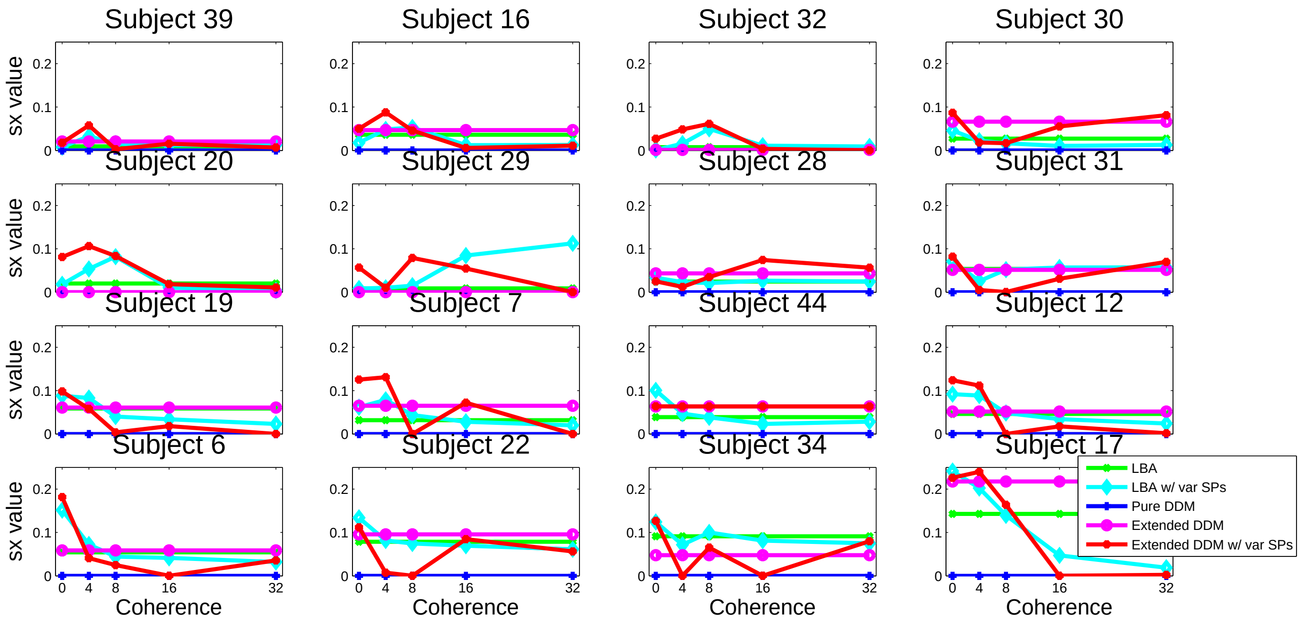

FIGURE 12. Comparison of variability in starting point model fits, by participant and closeness to OPC for the DDM. On average, variability appears to be greater at low than high coherences. LBA values A have been scaled down by a factor of 5000, to fit on the same axis. The pure DDM has zero variability in starting points.

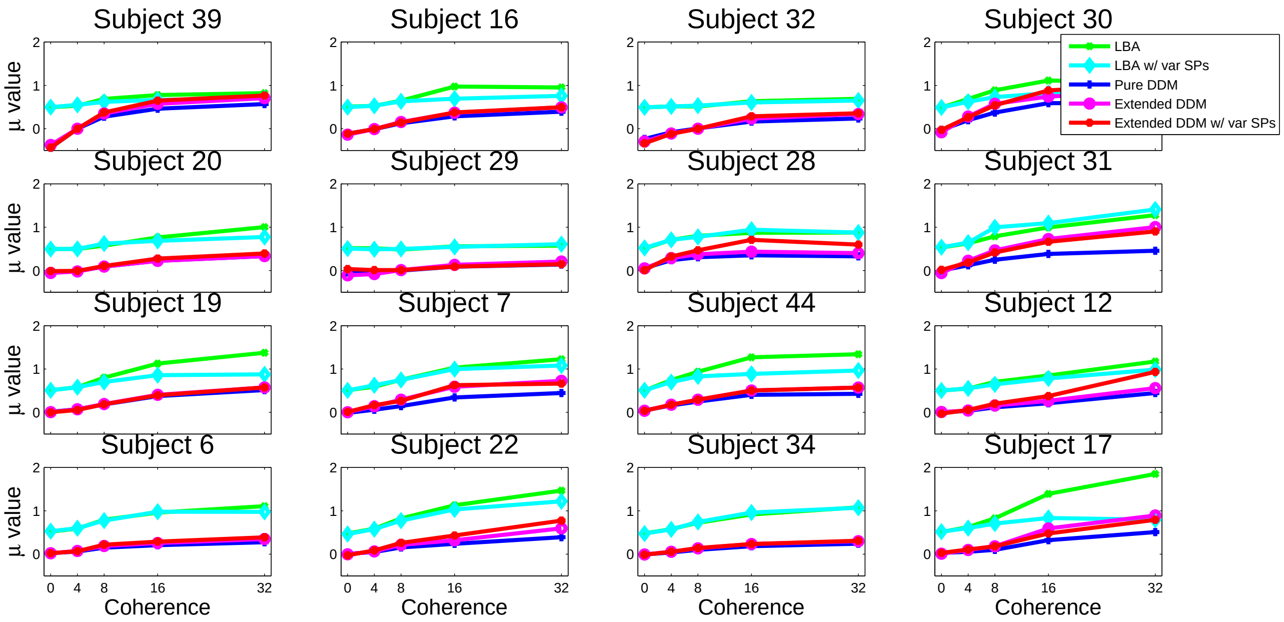

FIGURE 13. Comparison of mean drift parameter model fits, by participant and closeness to OPC for the DDM. As expected, all drift magnitudes increase monotonically with coherence. LBA drift rate estimates have not been shifted, as in the subject-averaged data of Figure 6(b).

(S. Goldfarb) HRL Laboratories, Malibu CA
E-mail address: segoldfarb@hrl.com

(N.E. Leonard) Mechanical and Aerospace Engineering, Princeton University, Princeton NJ
E-mail address: naomi@princeton.edu

(P. Simen) Neuroscience, Oberlin College, Oberlin OH
E-mail address: psimen@oberlin.edu

(C. Caicedo-Núñez) Mechanical and Aerospace Engineering, Princeton University, Princeton NJ
E-mail address: carlis.caicedo@gmail.com

(P. Holmes) Mechanical and Aerospace Engineering, Applied and Computational Mathematics and Neuroscience Institute, Princeton University, Princeton NJ
E-mail address: pholmes@math.princeton.edu
